# Supplementary material for: Nature of strong hole pairing in doped Mott antiferromagnets
Source: Sci Rep. 2014 Jun 24;4:5419. doi: 10.1038/srep05419 (PMC4067615; doi:10.1038/srep05419)
Supplement: Supplementary Information — for: Nature of strong hole pairing in doped Mott antiferromagnets [file srep05419-s1.pdf]

Supplementary Information for:
Nature of strong hole pairing in doped Mott antiferromagnets

Zheng Zhu, Hong-Chen Jiang, D. N. Sheng & Zheng-Yu Weng

In this supplemental material, we present some technical details. As an unbiased and powerful numerical tool to study strongly correlated systems in one dimensional and quasi-one dimensional ladder systems, the DMRG method is not limited by the exponential difficulty of total sites in exact diagonalization (ED) or the sign problem in quantum Monte Carlo (QMC) simulations. Since the ladders, serving as a bridge from one dimensional chains to two-dimensional layered systems, can be handled numerically to large sizes, they offer a chance to study the interplay of charge and spin degrees of freedom in the strongly correlated t-J model. Moreover, a Heisenberg ladder with even number of legs has a short-ranged spin correlation and a finite energy gap in magnetic excitations,[1] and the corresponding ground state may be regarded as the short-ranged RVB state proposed by Anderson,[2]. It may then lead to the hole pairing and superconductivity when holes are injected into the system as envisaged in Ref. [2].

The binding energy of the two-hole-doped t-J model is defined as $E_b = (E_2 - E_0) - 2(E_1 - E_0)$. For the present DMRG simulation, doping two holes is realized by taking two electrons out of the half-filling, and the state of the total spins can be either singlet with $S^z_{tot} = 0$ (removing an up spin and a down spin) or triplet with $S^z_{tot} = 1$ (removing two down spins). For the odd-leg ladders, we have shown the results of the singlet state in the main text, where one can see that there is no strong binding when $S^z_{tot} = 0$. Here in Fig. S1, the binding energies of the 1-leg and 3-leg t-J ladders in the triplet channel are shown. The binding energy is vanishingly small for different values of t/J in the thermodynamic limit, where the spin excitations behave like free spinons with a power-law decay of the spin-spin correlation length.

Based on the results of the binding energy E_b calculated at finite-length ladders, we can extrapolate the data to thermodynamic limit (i.e., $N_x = \infty$) according to a general fitting form $E_b(N) = E_b(N = \infty) + \frac{a}{N} + \frac{b}{N^2}$. Figure S2 shows the extrapolation of the binding energy for the two-hole-doped t-J ladders (with $N_y = 1, 3$) in both singlet channel ($S^z_{tot} = 0$) and triplet channel ($S^z_{tot} = 1$) at $t/J = 7$, which approaches vanishingly small binding energy at $N_x \to \infty$. Similarly, Fig. S3 shows the extrapolation of the binding energy for two-hole-doped $\sigma \cdot t$-J ladders ($N_y = 2, 3$) with $S^z_{tot} = 0$ at $t/J = 7$, which also indicates that no bound state exists in the thermodynamic limit. Figure S4 further compares the contrasting results of two different extrapolations, which illustrates the importance to calculate sufficiently long ladders in order to capture the right results in the thermodynamic limit, indicating that a finite-size scaling based on small clusters may lead to wrong extrapolations.

[1] E. Dagotto, T. M. Rice, Surprises on the Way from One- to Two-Dimensional Quantum Magnets: The Ladder Materials. *Science* **271**, 618 (1996).

[2] P. W. Anderson, The resonating valence bond state in La_2CuO_4 and superconductivity. *Science* **235**, 1196 (1987).

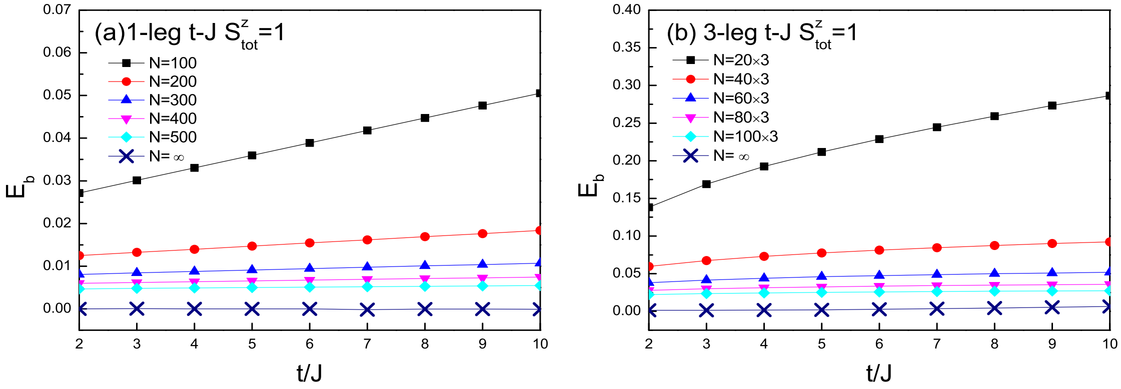

Fig. S1: (Color online) Two-hole binding energy E_b in a triplet ($S^z_{tot} = 1$) state : (a) 1-leg t-J chain; (b) 3-leg t-J ladder. The binding energy in the thermodynamic limit with $N_x \to \infty$ is vanishingly small according to the finite size scaling using second-order polynomials of $1/N$.

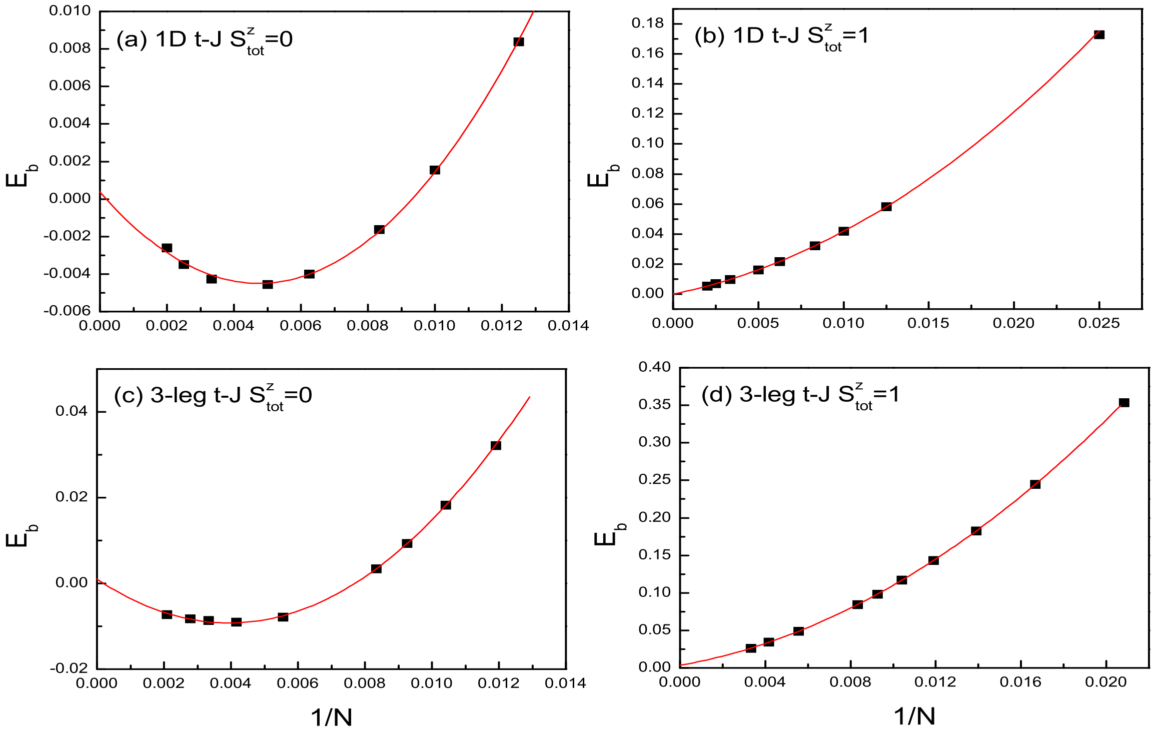

Fig. S2: (Color online) The extrapolation of the binding energy for the two-hole-doped t-J ladders at $t/J = 7$: (a) 1-leg with $S^z_{tot} = 0$; (b) 1-leg with $S^z_{tot} = 1$; (c) 3-leg with $S^z_{tot} = 0$; and (d) 3-leg with $S^z_{tot} = 1$. We extrapolate the results to the thermodynamic limit according to a general form $E_b(N) = E_b(N = \infty) + a/N + b/N^2$. The results show that the binding energy is almost zero when $N \to \infty$.

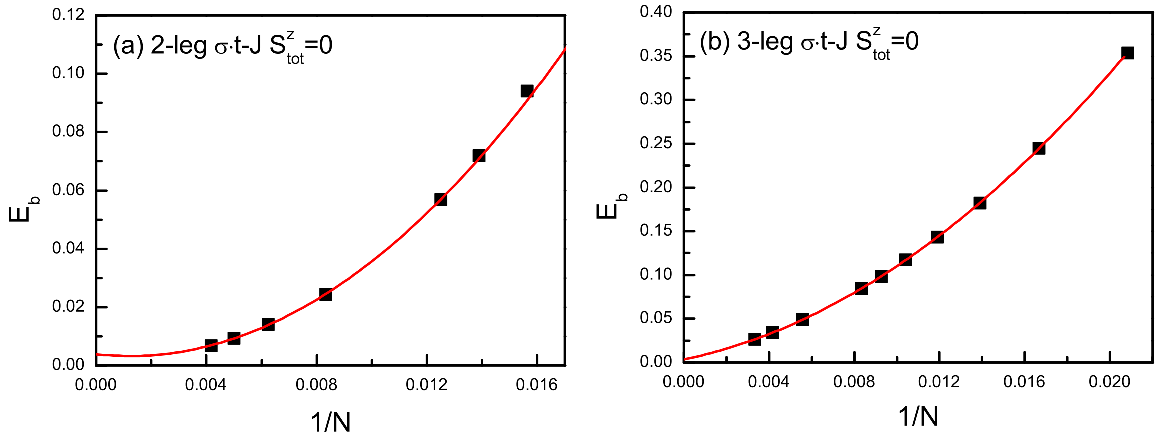

Fig. S3: (Color online) Typical examples of the extrapolation for the binding energy of two-hole-doped $\sigma \cdot t$-J ladders at $t/J = 7$: (a) 2-leg with $S^z_{tot} = 0$; (b) 3-leg with $S^z_{tot} = 0$. The extrapolation is made by using a general form $E_b(N) = E_b(N = \infty) + a/N + b/N^2$. The binding energy disappears at $N \to \infty$.

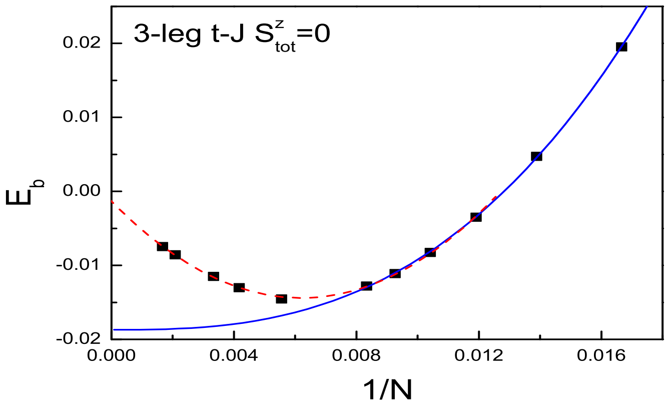

Fig. S4: (Color online) Typical examples of the extrapolation of the binding energy for two-hole-doped 3-leg t-J ladders at $t/J = 3$ with $S^z_{tot} = 0$. The blue solid line shows that if one only uses the data of smaller sizes for a finite-size scaling, it could lead to a wrong conclusion at $N_x \to \infty$. The correct extrapolation according to $E_b(N) = E_b(N = \infty) + a/N + b/N^2$, as presented by the red dashed line, shows that the binding energy is actually vanishingly small at $N = \infty$.
